# Presence of isthmi in mandibular mesial roots and associated factors: an in vivo analysis

**DOI:** 10.1007/s00276-019-02231-w

**Published:** 2019-04-01

**Authors:** Xiaoli Hu, Zijing Huang, Zhuwei Huang, Lizhen Lei, Minyi Cui, Xiaolei Zhang

**Affiliations:** 1 Department of Operative Dentistry and Endodontics, Guanghua School of Stomatology, Hospital of Stomatology, Guangdong Province Key Laboratory of Stomatology, Sun Yat-sen University, 56 Ling Yuan Xi Road, Guangzhou, 510055 Guangdong China; 2 Department of Radiology, Guanghua School of Stomatology, Hospital of Stomatology, Guangdong Province Key Laboratory of Stomatology, Sun Yat-sen University, Guangzhou, Guangdong China

**Keywords:** Root canal anatomy, Isthmus, Middle mesial canal, Mandibular first molar, Cone-beam computed tomographic

## Abstract

**Purpose:**

To investigate the prevalence of isthmi and middle mesial (MM) canals in the mesial roots of mandibular first molars (MFM) in a Mongoloid subpopulation and to evaluate their association with demographic and anatomic characteristics.

**Methods:**

Cone-beam computed tomography (CBCT) images of 496 patients with 823 MFMs were selected and analyzed. The following data were collected: patient age and gender, side, presence and distribution of MM canal and isthmus, distance between mesiobuccal (MB) and mesiolingual (ML) orifices, and MB–ML root canal system (RCS) morphology. Logistic regression was used to determine the association between demographic and anatomic characteristics and the presence of isthmi in the apical third.

**Results:**

The overall prevalence of isthmus and an MM canal in MFM was 64.6% and 10.8%, respectively. The highest prevalence of isthmi and MM canals was found in patients of ≤ 20 and of 41–60 years, respectively (*p* < 0.05). The prevalence of isthmi declines with age. A total of 41.3% of the MFMs had isthmi in the apical third of the mesial roots. Younger age, shorter MB–ML orifice distance, and Weine type II RCS increased the probability of the presence of an isthmus in the apical third (*p* < 0.05).

**Conclusion:**

The prevalence of isthmus in MFM is high in the subject population, but the prevalence of MM canals is not as high as previously reported. Demographic and anatomic characteristics could aid clinicians to better predict the presence of MM canal and an isthmus.

## Introduction

Thorough removal of microorganisms is one of the key factors for a successful endodontic treatment [19]. Although the dental operating microscope is nowadays widely used in nonsurgical endodontic treatment, missing canals and anatomical complexities are still common reasons for endodontic failure in mandibular molars [18].

The anatomical variation in the mesial roots of mandibular first molars (MFMs) includes an isthmus [6] and a middle mesial (MM) canal [16]. An isthmus is usually defined as a narrow pulp space that extends from two main root canals [13]. An MM canal has been described as a third canal between the mesiobuccal (MB) and mesiolingual (ML) canals in the mesial root [16]. Many methods such as staining and clearing, sectioning, and micro-computed tomographic (micro-CT) scanning have been used to study the root canal configuration of mandibular molars [5–7, 21]. Although these methods are technically feasible, it can be disputed that the extracted teeth are biased samples that might not represent healthy teeth in the general population. Cone-beam computed tomography (CBCT) is the tool of choice to study the internal root canal anatomy with high-resolution three-dimensional imaging, while lessening the superimposition and distortion of the neighboring anatomic structures. Moreover, as a noninvasive and reliable medical imaging technique, the relatively shorter scan time and less radiation allow CBCT to be employed for clinical applications and in vivo dental anatomy studies [9, 12].

Previous studies have reported complex root canal configurations of MFM along with demographic features including age, sex and race [6, 23]. Although micro-CT studies found that the prevalence of an isthmus in the mesial roots of the extracted MFMs was high in a Chinese population, these studies comprise very limited samples (*N* = 36 and *N* = 70, respectively) [5, 6]. Very little work has been explored on the prevalence and distribution of isthmus and MM canal in the mesial roots of MFMs in a large Mongoloid population in vivo.

A major reason for root canal treatment (RCT) failure in the mesial roots of molars is improper management or neglect of the isthmus [10]. During nonsurgical RCT or retreatment, clinicians often encounter challenges in recognizing the presence of an isthmus and the diagnosis of the etiology of previous treatment failure. Since CBCT is not a technique in the standard of care for routine endodontic diagnosis or screening in the absence of special clinical circumstances, the identification of an isthmus and its morphologic features in the apical portion is still impossible on two-dimensional periapical radiograph or by visual inspection under the dental operating microscope during nonsurgical RCT. Until now, no study has proposed clinical indicators to predict the presence an isthmus in the apical third of MFMs, which require more elaborate techniques to achieve therapeutic goals and to estimate the prognosis.

Therefore, the purposes of this in vivo study were the following:

1. To determine the prevalence and distribution of isthmi and MM canals in MFMs in a large Chinese population in vivo and to evaluate the related factors;
2. To analyze the association between the presence of isthmi in the apical third and the demographic and anatomic characteristics in the mesial root of MFMs.

## Materials and methods

### Subjects

The present study was reviewed and approved by the Ethics Committee of the Affiliated Stomatological Hospital of Sun Yat-sen University, Guangzhou, China (ERC-2017-09). Digitized CBCT of MFMs was randomly selected from archived images of patients who had taken CBCT for purposes of diagnosis before treatment at the Affiliated Stomatological Hospital, Sun Yat-sen University, between January 2010 and January 2018.

A sum of 496 patients having 823 MFMs were included in this study. Inclusion criteria were adapted from previous studies [4, 11]:

1. Patients with ages between 12 and 70 years;
2. MFMs with fully matured apices but without periapical lesions, root resorption or fractures;
3. Teeth without intraradicular fillings or any types of fixed prosthesis;
4. CBCT images of high quality.

### Radiographic techniques

All CBCT images were obtained using a CBCT scanner (DCTPRO, VATECH, Yongin-Si, Republic of Korea) with a field of view of 16 × 7 cm and a voxel size of 0.16 mm. The operating parameters were set at 90.0 kV and 9 mA with a scanning time of 24 s. The measurements were evaluated using Ez3D 2009 software.

### Radiographic evaluation

All images were carefully observed in three planes (axial, coronal, and sagittal) by an endodontist and an oral radiologist using the same criteria. When disagreement occurred, another endodontist reviewed the image until final consensus was reached. The intraobserver and interobserver reliability was assessed by calculating the kappa values, which were 0.815 (intraobserver) and 0.805 (interobserver).

In the axial view, when a narrow transverse connection between the MB and ML canal was visualized, an isthmus was recorded. The MM canal was recorded when an obvious circular radiolucency was detected between the MB and ML canals at axial plane despite whether an isthmus presented or not [13, 20]. When MM canals and isthmi were identified, their distributions were recorded according to their occurrence in different axial slices. The distributions were allocated into six classifications according to the location of the beginning and ending of the MM canal or isthmus. The six classifications were as follows: confined to cervical third, cervical third to middle third, cervical third to apical third, confined to middle third, middle third to apical third and confined to apical third [4]. The following data were gathered for further analysis:

1. Patients’ age and gender. Age was arbitrarily categorized into four groups: ≤ 20 years, 21–40 years, 41–60 years, and > 60 years;
2. Number of root and root canal in mesial roots;
3. The presence of isthmus and MM canal;
4. The location of the beginning and end of isthmi and MM canals;
5. The distance between MB and ML root canal orifices;
6. The canal configurations of mesial roots based on Weine’s classification. Here, Weine type I stands for single canal, Weine type II means that MB and ML canals end in one apical foramen while Weine type III demonstrates separated MB and ML apical foramina [25].

### Statistical analysis

Statistical analysis was accomplished using the Statistical Package for Social Sciences version 20 (SPSS, Chicago, IL, USA). Differences in the prevalence of isthmi and MM canals according to gender, age, and sides (left or right) were compared using Chi square tests. Differences in MB–ML orifice distances were compared by independent samples *t* test. Chi square tests and logistic regression were used to identify the association between the demographic and anatomic characters (independent variables), and the presence of an isthmus in the apical third (the outcome variable). The significance level was set at *p* < 0.05.

## Results

### Prevalence of MM canal and isthmi and related factors

**Fig. 1:**
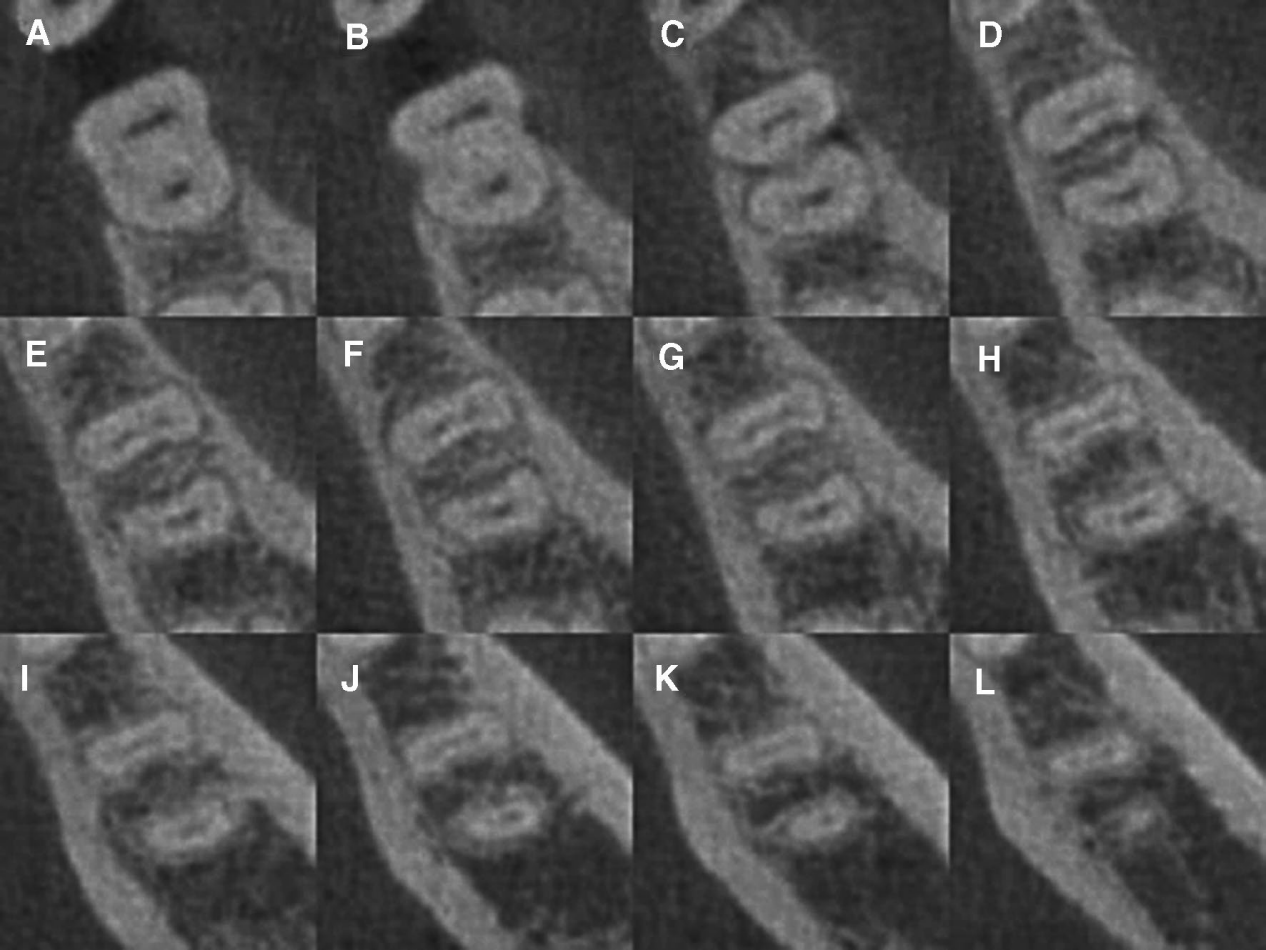
CBCT images showing the isthmus at different axial view levels of the mandibular first molar (tooth #30, Weine type III configuration) (coronal, middle, and apical). **a**–**d** Axial view of the mandibular first molar, reoriented to the long axis of the tooth to demonstrate the presence of isthmus, cervical section, **e**–**h** middle section, and **i**–**l** apical section

Of the 496 patients, 50.2% were men and 49.8% were women, with an average age of 38.4 years. The overall prevalence of isthmi was 64.6% (532/823) in the mesial roots. The distribution of isthmi in the mesial roots according to gender, age, and side is presented in Table 1. With respect to the age, there was a significant difference among the four groups (*p* < 0.01), declining with age. Patients younger than 20 years had the highest prevalence of isthmi (82.8%), whereas those older than 60 years had the lowest (43.8%). There was no gender or side difference related to the prevalence of isthmi (Table 1, Fig. 1).

**Table 1:** Prevalence of isthmi/MM canals in mandibular first molars and its association with patient gender, age, and side (*n* = 823)

	Gender			Age (years)					Side			Total (%)
	Male (%)	Female (%)	*p*	≤ 20 (%)	21–40 (%)	41–60 (%)	> 60 (%)	*p*	Right (%)	Left (%)	*p*	
Isthmus			0.06					< 0.01			0.98	
With	254 (61.5)	278 (67.8)		53 (82.8)	308 (69.5)	143 (56.7)	28 (43.8)		261 (64.6)	271 (64.7)		532 (64.6)
Without	159 (38.5)	132 (32.2)		11 (17.2)	135 (30.5)	109 (43.3)	36 (56.2)		143 (35.4)	148 (35.3)		291 (35.4)
MM canal			0.16					< 0.01			0.63	
With	51 (12.3)	38 (9.3)		6 (9.4)	36 (8.1)	42 (16.7)	5 (7.8)		43 (10.6)	49 (11.7)		89 (10.8)
Without	362 (87.7)	372 (90.7)		58 (90.6)	407 (91.9)	210 (83.3)	59 (92.2)		361 (89.4)	370 (88.3)		734 (91.2)
Total	413	410		64	443	252	64		404	419		823

*MM canal* middle mesial canal; data were analyzed by Chi square test

**Fig. 2:**
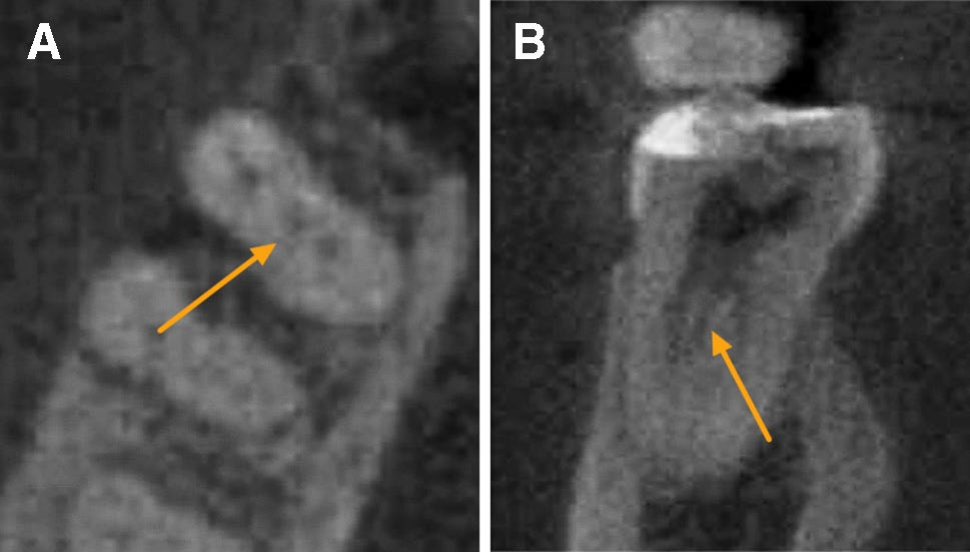
CBCT images showing the middle mesial canal of the mandibular first molar (tooth #30) in the axial (**a**) and coronal (**b)** plane. The arrows point to the middle mesial canal

Of the 823 MFMs, 89 (10.8%) were found with MM canals. The Chi square test showed that the factor “age” was associated with the prevalence of MM canals (*p* < 0.01), while the prevalence of MM canals was not related to the other two factors “gender” and “side” (*p* > 0.05). The prevalence of MM canals was 16.7% in the age group of 41–60 years, which was significantly higher than in those aged 21–40 years (*p* < 0.01; Table 1). The prevalence of MM canals in the 41–60 year group was also higher than in the other two age groups (≤ 20 and > 60 years), but the differences were not statistically significant (*p* > 0.05; Table 1, Fig. 2).

### Distribution of isthmi and MM canals according to the location

The distribution of the isthmi and MM canals according to the beginning and end are shown in Table 2. In 532 MFMs with isthmi in mesial roots, 170 (20.6%) and 4 (0.5%) were confined to the cervical and middle third, respectively; 340 (41.3%) had isthmi in the apical third; 169 (20.5%) had isthmi beginning from the cervical third with extensions going into the apical third; and 171 (20.8%) had isthmi beginning in the middle third and ending in the apical third or began and ended in the apical third.

**Table 2:** Distribution of isthmi and MM canals in mandibular first molars according to the location of the isthmus or the MM canal’s beginning and end (*n* = 823)

	Isthmus (%)	MM canal (%)
Confined to cervical third	170 (20.6)	22 (2.7)
Cervical third to middle third	18 (2.2)	21 (2.6)
Cervical third to apical third	169 (20.5)	7 (0.9)
Confined to middle third	4 (0.5)	24 (2.9)
Middle third to apical third	18 (2.2)	8 (1.0)
Confined to apical third	153 (18.6)	7 (0.9)
Total	532 (64.6)	89 (10.8)

*MM canal* middle mesial canal

Among the 89 MM canals identified, 29 had separate orifices, 2 shared the orifice with either the MB or ML canal but had their own apical foramen, and the rest diverged from either the middle or the apical third of the MB or ML canal. Only 3 of the 89 MM canals were independent canals with separate orifices and apical foramen.

### Presence of isthmi in the apical third and predictive factors

The associations between demographic and anatomic characteristics and the presence of an isthmus in the apical third are presented in Table 3. Among these factors, age, MB–ML isthmus (cervical third), the MB–ML orifice distance, and Weine’s classification were found to be associated with the presence of isthmi in the apical third (*p* < 0. 01; Table 3). The prevalence of isthmi in the apical third in age groups of ≤ 20 years, 21–40 years, 41–60 years, and > 60 years was 59.4% (38/64), 44.2% (196/443), 36.1% (91/252), and 23.4% (15/64), respectively, meaning the presence of isthmi in the apical third decreased with age. Among MFMs with isthmi in the apical third, 169 (49.7%) presented isthmi in the cervical third. In MFMs without isthmus in the apical third, 188 (38.9%) had isthmi in the cervical third (*p* < 0.01). The mean MB–ML orifice distance was 2.82 mm in MFMs in which the isthmus was recognized, whereas the mean distance was 2.95 mm in those without isthmus (*p* < 0.01).

**Table 3:** Associations between demographic/anatomic characteristics and the presence of the isthmus in the apical third

Variable^a^	MFM with isthmus in the apical third (*n* = 340, %)	MFM without isthmus in the apical third (*n *= 483, %)	*p* value
Age			< 0.01
≤ 20 years	38 (11.2)	26 (5.4)	
21–40 years	196 (57.6)	247 (51.1)	
41–60 years	91 (26.8)	161 (33.3)	
> 60 years	15 (4.4)	49 (10.1)	
MB–ML isthmus (cervical third)			< 0.01
Present	169 (49.7)	188 (38.9)	
Absent	171 (50.3)	295 (61.1)	
Presence of MM canal			0.86
Yes	36 (10.6)	53 (11.0)	
No	304 (89.4)	430 (89.0)	
MB–ML orifice distance, mean	2.82 mm	2.95 mm	< 0.01
Weine configuration of the mesial root canal system (MB and ML)^b^			< 0.01
Type II	127 (37.4)	6 (1.3)	
Type III	213 (62.6)	474 (98.8)	

*MFM* mandibular first molar, *MM canal* middle mesial canal, *MB* mesiobuccal, *ML* mesiolingual

^a^The independent variables “age,” “presence of isthmus in cervical third,” “presence of MM canal between the MB and ML canals,” and “Weine configuration of the mesial root canal” were considered as categorical variables in the Chi square analysis for the association between these predictor (independent) variables and the presence of the isthmus in the apical third (outcome variable). “MB-ML orifice distance” was considered as a continuous variable and analyzed by the independent samples *t* test

^b^Since three mandibular first molars with Weine type I configuration (single canal) in mesial roots were excluded, 820 mandibular first molars were categorized into Weine type II or II configuration

In MFM mesial roots with isthmi in the apical third, 37.4% were Weine type II configuration and 62.6% were Weine type III. Nearly all Weine type II MFMs (127/133) had isthmi in the apical third. The presence of isthmus was about 30% (213/687) for Weine type III MFM (*p* < 0.01). The presence of isthmi in the apical third was not related to the presence of MM canals (*p* > 0.05).

In the logistic regression model, the age, MB–ML Weine configuration and MB–ML orifice distance of the mesial root canal system were significantly related to the presence of isthmus in the apical third (*p* < 0.05) while MB–ML isthmus (cervical third) was no longer associated with isthmus in the apical third (*p* > 0.05) (Table 4). Patients younger than 20 years were about eight times more likely to have an isthmus in the apical third, compared to patients older than 60 years (*p* < 0.01, odds ratio [OR] = 7.62). Patients between 21 and 40 years of age and between 41 and 60 years of age were about four times (*p* < 0.01, OR = 3.84) and two times (*p* < 0.05, OR = 2.45) more likely to have an isthmus in the apical third, respectively. The shorter the distance between the MB–ML orifices, the more likely did an isthmus present in the apical third (*p* < 0.05, OR = 0.67). In other words, the presence of an isthmus was about two times more likely to be identified in MFM with every 1-mm decrease in the distance of MB–ML orifices. A significant association was found between the MB–ML Weine’s configuration and the existence of an isthmus in the apical third. Weine type II mesial roots were almost 51.95 times more likely to present an isthmus in the apical third than the Weine type III molars (*p* < 0.01, OR = 51.95).

**Table 4:** Logistic regression analyses of predictor variables as factors related to the presence of isthmus in the apical third

Independent variables^a^	Adjusted OR (95% CI)	*p* value
Age		
**≤ **20 years (> 60 years as reference)	7.62 (2.95–19.68)	< 0.01
21–40 years (> 60 years as reference)	3.84 (1.72–8.60)	< 0.01
41–60 years (> 60 years as reference)	2.45 (1.07–5.62)	0.03
MB–ML orifice distance	0.67 (0.46–0.98)	0.04
Weine configuration of MB and ML		
Type II (type III as reference)	51.95 (22.13–121.99)	< 0.01
Isthmus cervical (absent as reference)	1.25 (0.89–1.75)	0.19

*OR* odds ratio, *CI* confidence interval, *MM canal* middle mesial canal, *MB* mesiobuccal, *ML* mesiolingual

^a^The independent variables “age” and “Weine configuration of the mesial root canal” were considered as categorical variables, with “MB-ML orifice distance” being a continuous variable in the logistic regression analysis for the association between these predictor (independent) variables and the presence of the isthmus in the apical third (outcome variable)

## Discussion

Using in vivo CBCT, this study investigated the configuration of the mesial roots of 823 MFMs with a sample size 10 times larger than in previous studies [2, 3, 6, 20].

In this study, the overall prevalence of MM canals was 10.8% in the mesial roots of MFMs, which was much lower than the prevalence reported as 26% in an American population [20]. The prevalence of MM canals was detected to be significantly higher in a Brazilian (22.1%) compared with a Turkish population (14.8%), which suggested that MM canals in MFMs might be related to race [23]. The overall prevalence of isthmi in our study was 64.6%, similar to other in vivo studies, which reported a frequency of isthmi from 57.8% to 83% in the mesial roots of MFMs, respectively [4, 24].

Our study presented that the prevalence of isthmus in the apical third was 41.3% (340/823), which was similar to other in vivo CBCT studies [4, 14, 20]. A frequency of 83% for isthmi was reported by endoscopic inspection during root end surgeries [24]. The sample in that study consisted of root canal-treated teeth associated with pathological lesions. Hence, it might be a biased sample, as teeth with untreated isthmi during RCT are more likely to fail and present for surgery. Compared with in vivo studies, in vitro studies presented a much higher prevalence of isthmus in the apical third. Two in vitro micro-CT studies [5, 6] reported a similar prevalence of isthmus in the apical portion of the mesial roots in MFMs (88.9% and 86.0%, respectively). Considering that those studies were based on extracted teeth of relatively small sample size (*N* = 36 and 70, respectively), our study with a large sample of vital teeth would likely provide a more reliable estimate.

Age was the factor associated with the presence of both isthmi and MM canals in the mesial roots of MFMs. The prevalence of isthmi declined as age increased, and the prevalence of MM canals was significantly higher in the age group of 41–60 years, which is in line with the results of other studies [6, 20]. As is widely accepted, root canal morphology changes with teeth development, laying down secondary dentine, and maturing. Peiris et al. [15] studied the root canal morphology changes with ages. The mesial roots of MFMs demonstrated only one large canal before 11 years old. Over time, secondary dentine deposited in a mesiodistal direction in root canals results in canals dividing from one large canal into two separated canals. Further deposition of secondary dentine with age makes the root canal system more complex, but intercanal communications remain. Meanwhile, with even further advances in age and further deposition of secondary dentine, intercanal communication may disappear, and two narrow, separated canals ensue. Therefore, intercanal communications were low in the younger and older age groups but high in the intermediate age groups [15]. This may explain the declining prevalence of isthmi with age and the higher prevalence of MM canals in the age group of 41–60 years in our study. Younger age was also positively associated with the presence of isthmi in the apical third in the logistic regression analysis. During nonsurgical RCT, the presence of isthmi and MM canals may have an impact on effective debridement and will likely increase the rate of treatment failure. Ricucci et al. [17] found that the success rates of endodontic treatment increased with age. When treating younger patients, more attention should be paid to disinfect the root canal system thoroughly. This also points to the need to be more conservative during treatment planning when managing deep carious lesions in younger patients because of the relatively lower success rates of RCT in the younger group.

MB and ML anatomic characteristics, such as MB–ML orifice distance as a continuous variable and Weine configuration of mesial roots as a categorical variable, were found to be associated with the presence of isthmi in the apical third in logistic regression analysis. The presence of an isthmus in the apical third was two times more probable to be identified in MFM with every 1-mm decrease in distance of MB–ML orifices. In our study, mesial roots with Weine type II configuration, in which MB and ML canals ended in one apical foramen, were more likely to have isthmi in the apical third, showing that most Weine type II MFMs (127/133) had isthmi in the apical third. When a root resection is necessary in type II cases, the unfilled canal or the isthmi should be instrumented and filled to reduce the failure of root end surgery [10]. Weine type III MFMs (687/823) were much more common than Weine type II MFMs (133/823) in this study. While the presence of isthmus was almost universal in Weine type II canals, Weine type III MFMs contributed 62.6% of all MFMs having isthmi in the apical third. Since it may be difficult for clinicians to identify such root canal configurations clinically, they are encouraged to assume the presence of either Weine’s type II or III canal [25]. In the logistic regression model, MB–ML isthmus (cervical third) was not associated with isthmus in the apical third. In addition, 20.8% (171/823) and 1.9% (15/823) of MFMs had root canal isthmus and MM canal beginning in the middle third or apical third and ends in the apical third in the present study. While mechanical instrumentation and irrigation of the main canals and isthmi commencing from the cervical area are relatively straightforward, the access to those canals when root canal isthmi and MM canals begin in the middle or apical third is challenging.

In consideration of the complex anatomy of the mesial roots in MFM, it is necessary to meticulously instrument and thoroughly irrigate root canals during orthograde RCT, to improve the chances of any irrigants going into the isthmi. During retrograde preparation, the proper identification of any isthmus with an operating microscope, including it in the retrograde preparation, and filling of the prepared cavity thereafter are critical steps in surgical endodontics. Passive ultrasonic irrigation, EndoVac, and laser-activated irrigation techniques have been used to improve the chemical effects of irrigation solutions since they effectively remove smear and debris from the complex root canal system [1, 8, 22]. Therefore, when treating MFMs, especially mesial root canals, special and effective irrigation should be considered as a routine procedure. Nevertheless, none of the existing irrigation protocols was able to free mandibular molars completely from packed debris [8]. Further research is necessary to improve current irrigation protocols. Besides a rigorous regimen of irrigation, intracanal dressing has also been favored to improve microbial reduction and to cope with the challenges related to anatomic complexities and with root canal preparation procedures [4].

In conclusion, demographic and anatomic characteristics of the mesial roots could cue clinicians to the presence of MM canal and an isthmus. The prevalence of isthmi and MM canals in the Chinese population was 64.6% and 10.8%, respectively. The prevalence of isthmi was higher in the younger age groups, and the prevalence of MM canals was significantly higher in the age group of 41–60 years. Patients of younger ages, shorter MB–ML orifice distances, and Weine type II canals were highly likely to have isthmi in the apical third. In appreciation of these results, when managing a persistent infection of the mesial root of the MFM, clinicians should consider the use of additional imaging technology, such as CBCT, to better study the anatomy of the failing roots and achieve better therapeutic goals.
